# A correlation study of intestinal microflora and first‐episode depression in Chinese patients and healthy volunteers

**DOI:** 10.1002/brb3.2036

**Published:** 2021-05-07

**Authors:** Shaojun Zheng, Yubing Zhu, Weidong Wu, Qi Zhang, Yongqian Wang, Zhiren Wang, Fude Yang

**Affiliations:** ^1^ College of Basic Medical and Clinical Pharmacy China Pharmaceutical University Nanjing China; ^2^ Department of Pharmacology Basic Medical College Inner Mongolia Medical University Huhehaote China; ^3^ HuiLongGuan Clinical Medical School Beijing HuiLongGuan Hospital Peking University Beijing China

**Keywords:** depression, intestinal flora, intestinal–brain axis, OUT

## Abstract

**Objective:**

This research examines the intestinal‐associated flora of patients with depression compared with healthy volunteers to identify the characteristics and differences of flora associated with depression. It provides a theoretical basis for the prevention and treatment of depression through intestinal micro‐ecological regulation.

**Methods:**

We recruited 30 patients with depression to participate in the patient group (PG), and 30 volunteers were recruited for the healthy control group (HG) from the Beijing Hui‐long‐guan Hospital. Thereafter, the 16S rRNA high‐throughput sequencing method, using the Hamilton Depression Scale, was applied to analyze patient and healthy groups.

**Results:**

PG and HG microflora were different regarding phylum, Family, Genus, and Order. The results showed that *Barnesiella* was the dominant flora in depression patients, while Lachnospiraceae and *Alloprevotella* were the dominant bacteria in healthy participants. The proportion of *Betaproteobateria* (Proteobacteria), Alcaligenaceae (proinflammatory), Peptostreptococcaceae, *Catenibacterium*, *Romboutsia*, *Sutterella*, and Burkholderiales in the anxiety‐negative depressed group was significantly higher than in the anxiety‐positive group; and the proportion of *Anaerostipes* (inflammation) and *Faecalibacterium* (anti‐inflammatory) bacteria was significantly lower than that of patients with anxiety.

**Conclusion:**

Results showed there were differences in intestinal micro‐ecology between patients with depression and healthy volunteers. We found that the level of inflammation‐related bacteria in anxiety‐positive patients was lower than that in anxiety‐negative patients. These results enrich the knowledge of relationships between depression and intestinal flora and provide a theoretical basis for probiotics to assist in the treatment of depression.

## Graphical abstract

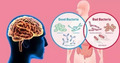

## 1 INTRODUCTION

The intestinal flora is a dynamic and complex microbial ecosystem which contains a number of genes magnitudes higher than humans. The adults have a weight of about 1 kg (Dinan et al., 2015), including bacteria, fungi, and viruses (Collins et al., 2009). At present, more and more evidence demonstrates that there is an intricate interaction between the host and the gut flora at almost all levels, from direct communication between cells to a wide range of signals among different systems, which involves various organs and body systems, including the central nervous system (Arumugam et al., 2011; Stilling et al., 2014).

Intestinal flora affects emotion and cognition through the gut–brain axis (Cryan & Dinan, 2012; Heijtz et al., 2011). The gut–brain axis is a two‐way neurohumoral conduction system. It contains a series of bidirectional pathways, including the hypothalamic–pituitary–adrenal axis (HPA), the vagus nerve, immune pathways, and metabolism (Bruce‐Keller et al., 2017; Dinan & Cryan, 2017). These complementary pathways promote interaction between the gut flora and the brain.

First, the intestinal flora can produce many neuroactive compounds (Dinan et al., 2013). For example, gamma‐aminobutyric acid (GABA) is secreted by lactic acid bacteria and *Bifidobacteria*, *Candida*, *Streptococcus,* and *Escherichia coli flora* all secrete 5‐hydroxytryptamine (5‐HT) in the intestinal (Barrett et al., 2012; Schousboe & Waagepetersen, 2007), and *Bacillus* and *Escherichia coli* secrete norepinephrine (NE) (Roshchina, 2010). Second, the vagus nerve plays an important role in signaling between the brain and the intestine. In addition, the immune system strengthens the relationship between the intestinal flora and the brain (Dinan et al., 2015). Levels of various inflammatory biomarkers have been shown to be elevated in depression patients (Raison & Miller, 2011). Additionally, infections of food‐borne campylobacter jejuni increased c‐reactive protein levels in brain regions associated with autonomic function and increased depression‐like behavior in mice. The dysfunction of the intestine–brain axis may cause physiological and pathological consequences (Mayer, 2011), and the abnormal function of the endocrine system plays an important role in the development of depression. Related studies show that (Sudo, 2014) intestinal flora can affect development and behavioral regulation. Therefore, the intestinal flora is a key node in the gut–brain axis and can provide a new target for the treatment of depression (Dinan & Cryan, 2017). This study aims to explore the changes of intestinal flora in patients with depression, to better understand its cause and development; second, to improve depression treatment by regulating intestinal flora to achieve a better prognosis, and try to prevent recurrence.

## 2 MATERIALS AND METHODS

### 2.1 Participants

This study is a controlled trial that was conducted at Beijing Hui‐Long‐Guan Hospital to explore changes in the intestinal flora of patients with depression. From November 2017 to February 2018, we included 30 outpatients and inpatients with depression for the patient group (PG) and 30 healthy participants (HG) for the control group.

Before inclusion in the study, we assessed the physical condition of the participants. The patients with depression were assessed using the Hamilton 24‐item depression scale and screened for the inclusion criteria in Table 1. We divided the experimental group into groups 1 and 2 according to the reference standard. See the results for details (Tables 2 and 3). Before the assessment scale, we conducted a scale consistency training for the researchers.

**TABLE 1:** Inclusion and Exclusion criteria

	Depression was diagnosed in patients meeting ICD‐10 criteria and treated for the first time
	Age 18–65 years old
Inclusion criteria	Blood routine, blood biochemistry, and routine examination without abnormalities
	Healthy control group without gastrointestinal disease clinically diagnosed
	Mental illness other than depression
	Took antibiotics four weeks prior to the study.
	Used contraceptives, nonsteroidal anti‐inflammatory drugs, laxatives/antidiarrhea drugs within the first two weeks of enrollment
Exclusion criteria	Changed eating habits within the first four weeks of enrollment
	Have taken antidepressants and other antipsychotic drugs in the past month
	History of abdominal surgery other than appendicitis
	Combined with serious physical illness
	Pregnancy or lactation in women

**TABLE 2:** HAMD score in the patient group

Number	HAMD Scale	Number	HAMD Scale
PG1	20	PG16	13
PG2	25	PG17	17
PG3	14	PG18	15
PG4	33	PG19	18
PG5	20	PG20	20
PG6	22	PG21	29
PG7	24	PG22	36
PG8	33	PG23	23
PG9	42	PG24	14
PG10	17	PG25	13
PG11	19	PG26	15
PG12	17	PG27	18
PG13	22	PG28	39
PG14	36	PG29	23
PG15	18	PG30	12

**TABLE 3:** The experimental group was divided according to anxiety scores (the first is PG1 and the second is PG2)

Number	HAMD scale	HAMA scale
PG9	42	41
PG28	39	38
PG22	36	36
PG14	36	28
PG23	23	25
PG26	15	24
PG27	18	24
PG8	33	23
PG13	22	21
PG21	29	21
PG29	23	19
PG6	22	18
PG30	12	17
PG25	13	16
PG4	33	15
PG11	19	14
PG7	24	13
PG12	17	13
PG17	17	13
PG18	15	13
PG20	20	13
PG2	25	12
PG5	20	12
PG1	20	10
PG19	18	10
PG10	17	9
PG3	14	8
PG15	18	8
PG24	14	8
PG16	13	6

### 2.2 Research methods

#### 2.2.1 Evaluation tool

The Hamilton Anxiety Rating Scale (HAMA) is one of the earliest commonly used scales in psychiatric clinical practice, including 14 projects. It is often used clinically for the diagnosis and classification of anxiety disorders. The Hamilton Depression Rating Scale (HAMD) is the most commonly used scale for clinically assessed depression. This study used the 24‐item version of the scale to evaluate the severity of the condition and the treatment effect. The assessment criteria are based on relevant information. Before conducting the measurement scale, we trained the researchers on consistency of the scale.

#### 2.2.2 Sample collection and sequencing

Approximately 2 g of a stool sample was collected from PG; it was placed immediately in a stool collection tube with 2 ml of preservation solution and frozen in a refrigerator at −80°C and standby application; the samples for HG were collected under the same conditions. Then, the 16S rRNA high‐throughput sequencing technology was used to analyze the differences in flora between the PC and HC groups (Claesson et al., 2011; Dethlefsen et al., 2008).

### 2.3 Build database and bioinformatics analysis process

#### 2.3.1 DNA extraction

Samples were stored at −80°C for DNA extraction. The DNA was extracted from 200 mg samples using the QIAamp DNA Stool Mini Kit (QIAGEN) following the manufacturer's instructions. DNA concentration and purity were checked by running the samples on 1.0% agarose gels.

#### 2.3.2 PCR amplification of 16S rRNA genes and Miseq sequencing

Polymerase chain reaction (PCR) amplification of 16S rRNA genes was performed using general bacterial primers (515F 5'‐GTGCCAGCMGCCGCGGTAA‐3' and 926R 5'‐CCGTCAATTCMT TTGAGTTT‐3'). The primers also contained the Illumina 5'overhang adapter sequences for two‐step amplicon library building, following manufacturer's instructions for the overhang sequences. The initial PCRs were carried out in 25 μl reaction volumes with 1‐2μl DNA template, 250 mM dNTPs, 0.25 mM of each primer, 1× reaction buffer, and 0.5 U Phusion DNA Polymerase (New England Biolabs). PCR conditions consisted of initial denaturation at 94°C for 2 min, followed by 25 cycles of denaturation at 94°C for 30 s, annealing at 56°C for 30 s, and extension at 72°C for 30 s, with a final extension of 72°C for 5 min. The second step PCR with dual 8‐base barcodes was used for multiplexing. Eight cycle PCR reactions were used to incorporate two unique barcodes at either end of the 16S amplicons. Cycling conditions consisted of one cycle of 94°C for 3 min, followed by eight cycles of 94°C for 30 s, 56°C for 30 s, and 72°C for 30 s, followed by a final extension cycle of 72°C for 5 min. Prior to library pooling, the barcoded PCR products were purified using a DNA gel extraction kit (Axygen) and quantified using the FTC ‐3000 TM real‐time PCR. The libraries were sequenced by 2*300 bp paired‐end sequencing on the MiSeq platform using MiSeq v3 Reagent Kit (Illumina) at Tiny Gene Bio‐Tech (Shanghai) Co., Ltd.

### 2.4 Bioinformatic analysis

The raw fastq files were demultiplexed based on the barcode. PE reads for all samples were run through Trimmomatic (version 0.35) to remove low quality base pairs using these parameters (SLIDING WINDOW: 50:20 MINLEN: 50). Trimmed reads were then further merged using FLASH program (version 1.2.11) with default parameters. The low‐quality contigs were removed based on screen.seqs command using the following filtering parameters, maxambig = 0, minlength = 200, maxlength = 580, maxhomop = 8. The 16S sequences were analyzed using a combination of software mothur (version 1.33.3), UPARSE (usearch version v8.1.1756, http://drive5.com/uparse/), and R (version 3.2.3). The demultiplexed reads were clustered at 97% sequence identity into operational taxonomic units (OTUs) using the UPARSE pipeline.

### 2.5 Ethical statement

The study has been approved by the Ethics Committee of Hui‐Long‐Guan Clinical Medical College, Peking University. All participants signed an informed consent form and a complete and comprehensive introduction for them or their Family which included the purpose, procedure, and possible risks of the study. Volunteers had the right to withdraw from the study at any time.

## 3 RESULT

The HAMD scores in the patient group and the division of the experimental group according to anxiety scores have been outlined in Tables 2 and 3, respectively.

### 3.1 Demographic data

The data represented by mean value, standard deviation, and comparison among groups, using independent sample *t* test, have been outlined in Table 4.

**TABLE 4:** Data are represented by mean value, standard deviation, and the comparison among groups which used independent sample *t* test

Groups	Male	Female	Total	*X^2^*/*P*
PG	12	18	30	0.591
HG	13	17	30	0.487

Groups	PG (*n* = 30)	HC (*n* = 30)	*t*	*p* < .05
Age (year)	30.80 ± 10.85	33.37 ± 7.02	−1.088	0.281
Height (cm)	167.00 ± 7.44	166.00 ± 7.08	0.533	0.596
Weight (Kg)	60.10 ± 8.36	63 ± 10.75	−1.167	0.248
HAMD score	20.17 ± 7.92	0.30 ± 0.79	13.674	0.000

### 3.2 Sequencing data

After 16S rRNA sequencing all sixty samples, a total of 1,777,341 raw gene sequences were selected. Then, we obtained 1,496,472 high‐quality gene sequences after optimization, with an average of 24,532 per sample. To decrease the quantity of gene sequences and prevent the sequence diversity being overestimated, we clustered the high comparability sequences into one OTU. After clustering, a total of 477 units of OTU were obtained. There were no significant differences between the two groups.

**FIGURE 1:**
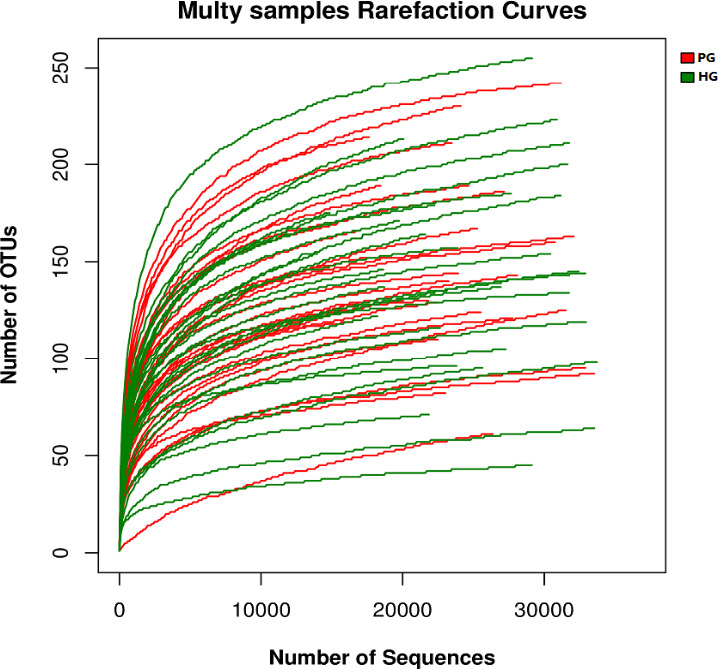
Rarefaction curve in the sample

Mothur software was used to draw a rarefaction curve to compare the richness of the flora in the sample (Figure 1). The rarefaction curve is a curve in which a certain number of individuals were randomly selected from the overall sample, and the number of Species represented by these individuals was counted and constructed by the number of individuals and Species. When the curve tends to be flat, this indicates that the sequencing is reasonable.

### 3.3 Abundance difference analysis

Microbial diversity is studied in community ecology. The diversity analysis (alpha diversity) of single samples can reflect the abundance and diversity of microbial communities, including a series of statistical analysis indices to estimate the Species abundance and diversity of environmental communities. Alpha diversity includes the Chao index, ACE index, Shannon index, Simpson index, and Sobe index. The Chao index and the ACE index reflect the Species richness of the sample, while the Shannon index and the Simpson index reflect the diversity of the Species, which is influenced by Species richness and Species evenness in the sample community. In the case of same Species richness, the greater the evenness of each Species in the community, the richer the diversity will be. The alpha diversity of this study is no significant differences such as Chao index, ACE index, Shannon index, Simpson index, and Sobe index between PG group and HG group.

### 3.4 Differential analysis of intestinal micro‐ecology

We found that the sequence of fecal bacteria mainly belongs to four phyla, *Bacteroidetes* (60.9%), *Firmicutes* (31.5%), *Proteobacteria* (5.9%), and *Fusobacteria* (0.8%). So, *Bacteroides* and *Firmicutes* account for about 92.4% of the total sequence in the fecal flora. The remaining sequences are classified as *proteobacteria*, *fusobacteria*, *actinomycetes*, etc. In addition, 0.9% of the unclassified bacteria are still unclassified.

At the level of Phylum, there were no statistically significant differences.

At the level of Class, the proportion of bacteria in *Epsilonproteobacteria* was significantly greater in PG (<0.1% versus < 0.1%, *p* = .012).

At the Family level, compared with the HG, the proportion of bacteria in Lachnospiraceae (14.4% vs. 9.8%, *p* = .003) and Succinivibrionaceae (0.5% vs. <0.1%, *p* = .019) was significantly lower in the PG; and Eubacteriaceae (<0.1% vs. < 0.1%, *p* = .003), Leuconostocaceae (<0.1% vs. <0.1%, *p* = .009), Campylobacteraceae (<0.1% vs. < 0.1%, *p* = .012), and Rikenellaceae (1.6% vs. 3.1%, *p* = .04) were significantly greater.

At the Genus level, the comparative analysis of the microbial community structure of the two groups showed that *Olsenella* (<0.1% vs. < 0.1%, *p* < .001), *Succinivibrio* (0.53% vs. <0.1%, *p* < .001), *Alloprevotella* (0.67% vs. <0.1%, *p* < .001), *Peptococcus* (<0.1% vs. <0.1%, *p* = .016), *Anaerostipes* (0.47% vs. 0.16%, *p* = .022), *Ezakiella* (<0.1% vs. <0.1%, *p* = .027%), and *Mogibacterium* (<0.1% vs. <0.1%, *p* = .032) were significantly lower in PG. *Cloacibacillus* (<0.1% vs. <0.1%, *p* < .001), *Asteroleplasma* (<0.1% vs. <0.1%, *p* < .001), *Anaerofilum* (<0.1% vs. <0.1%, *p* = .00116), *Eubacterium* (<0.1% vs. <0.1%, *p* = .0028), *Parvimonas* (<0.1% vs. <0.1%, *p* = .0031), *Weissella* (<0.1% vs. <0.1%, *p* = .009), *Proteus* (<0.1% vs. <0.1%, *p* = .012), *Campylobacter* (<0.1% vs. <0.1%, *p* = .012), *Alistipes* (1.54% vs. 3.09%, *p* = .024) showed a significant greater in the proportion of bacteria in HG.

At the Order level, the comparative analysis of microbial community structure between the two groups showed that Campylobacterales (<0.1% vs. <0.1%, *p* = .012) were significantly lower in PG, while Aeromonadales (0.54% vs. 0.084%, *p* = .018) were significantly greater in HG.

**FIGURE 2:**
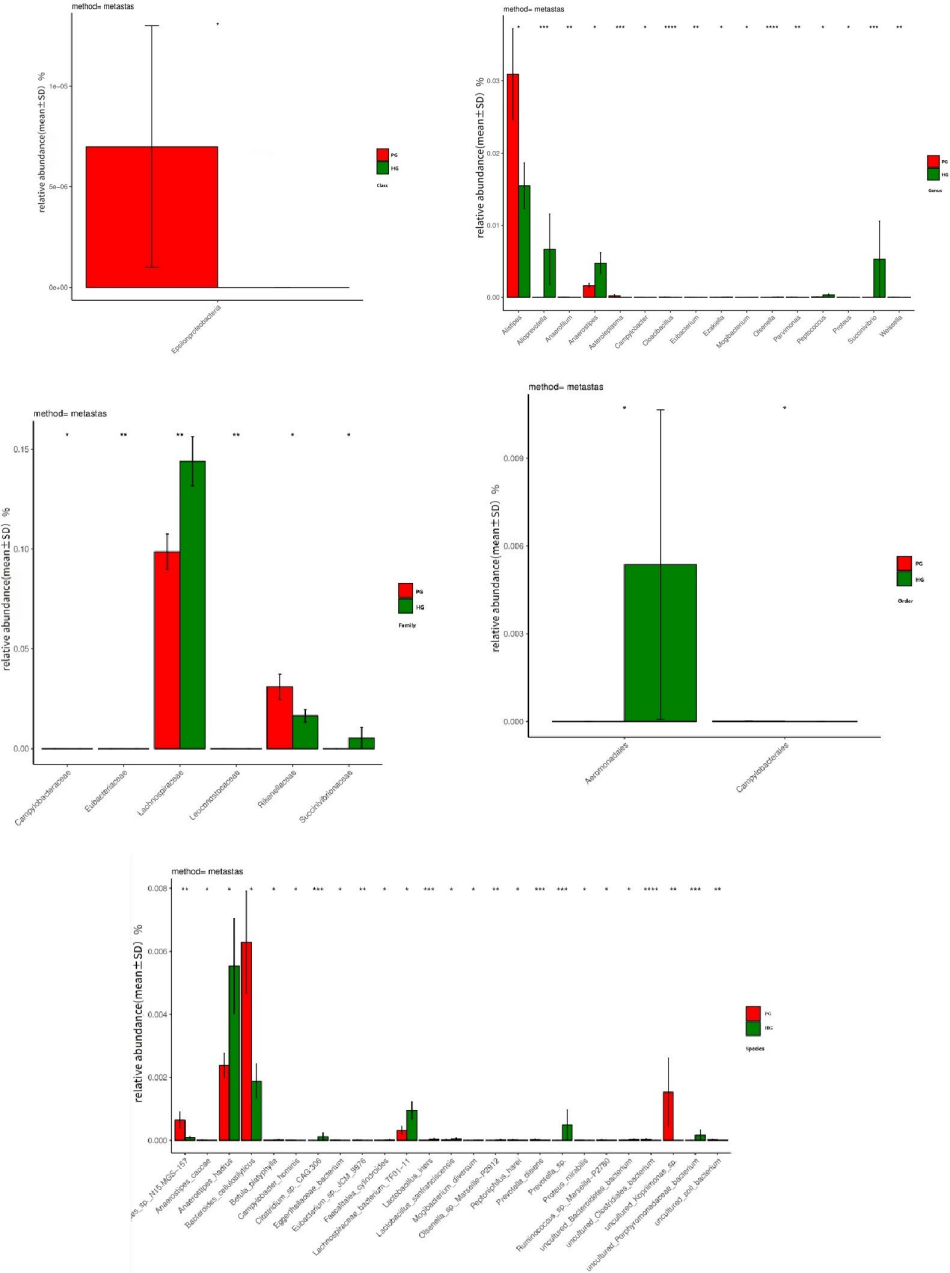
Differences at the level of Class, Family, Genus, Order, and Species; the abscissa is the Species of bacteria, and the ordinate is mean ± *SD*. * means it is relevant

Due to the complicated Species relationship between the two groups, the detailed results are shown in Figure 2 because the accuracy of the data is extremely low for bacterial groups with the relative abundance of less than 0.1% in MiSeq analysis. Therefore, we summarized the flora with abundance greater than 0.1%, as shown in Table 5.

**TABLE 5:** Detailed results of differential analysis between PG and HG

	OTU	Mean (PG)%	Mean (HG)%	*p*‐Value	*p*.signif
Family	Lachnospiraceae	0.09861	0.143887	.002997	**
	Succinivibrionaceae	<0.001	0.005365	.01998	*
	Rikenellaceae	0.030912	0.016479	.044955	*
Genus	Succinivibrio	<0.001	0.005294	.000999	***
	Alloprevotella	<0.001	0.006666	.000999	***
	Anaerostipes	0.001616	0.004733	.021978	*
	Alistipes	0.030912	0.015487	.023976	*
Order	Aeromonadales	<0.001	0.005365	.017982	*

Notes.

The relative abundance of 0.1% or more in at least one group,* indicates that it is relevant Multiple * indicates that it is more relevant.

### 3.5 The LDA Effect Size analysis of community differences between groups

**FIGURE 3:**
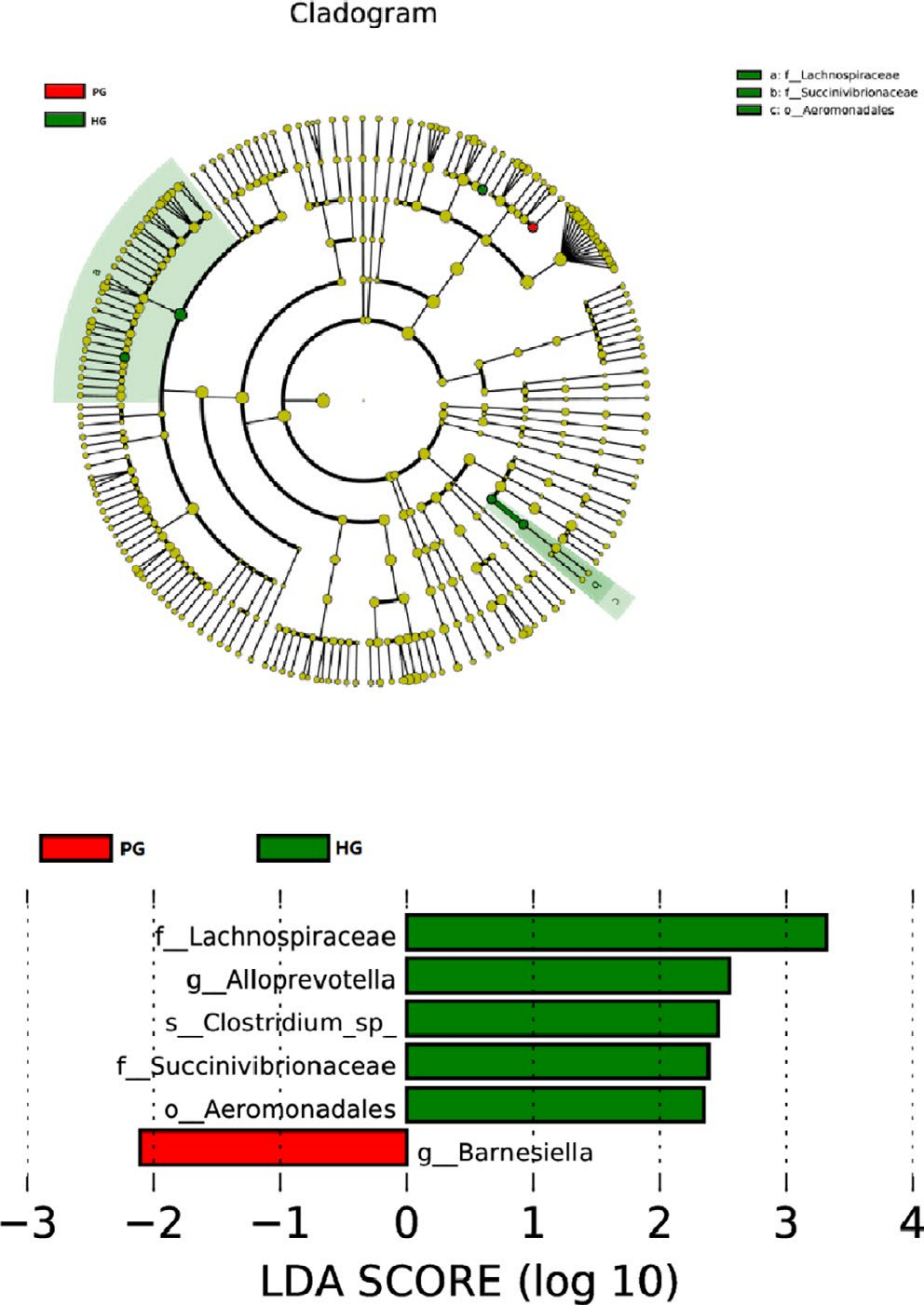
LEfSe tree graph and LDA fraction distribution. Onion parody of cluster tree, red and green areas represent different groups, the branches in the red nodes play an important role in the red group of microbial groups, green nodes play an important role in the green groups said the microbial groups, yellow nodes indicate microbial groups that have not played a significant role in two groups. Names of Species indicated by letters in the picture. This circle radiates from the inside out to the cluster tree, representing the classification levels of Phylum, Class, Order, Family, and Genus in turn. Each small circle on the different circle layers represents a classification at that level, and the diameter of the small circle is proportional to the relative abundance of the classification. The figure on the right is the LDA score obtained by LDA analysis (linear regression analysis) for the microbial groups with significant effects in the two groups

LEfSe is a software for discovering high‐dimensional biomarkers and revealing genomic characteristics, which includes genes, metabolism, and classification; it is used to distinguish two or more biological conditions (or groups). The algorithm emphasizes statistical significance and biological correlation. This allowed the researchers to identify the characteristics of different abundances and the associated categories. LEfSe has a strong recognition function through biological statistical differences. To be specific, the nonparametric factorial Kruskal‐Wallis (KW) sum‐rank test (nonparametric Kruskal‐Wallis and rank test) was first used to detect the characteristic of significant differences in abundance and find the groups with significant differences from the abundance. Finally, LEfSe used linear discriminant analysis (LDA) to estimate the effect of abundance of each component (Species) on the differential effect. According to the LDA SCORE, *Barnesiella* was the most dominant bacterium in the fecal microbial community of the experimental group (Figure 3).

### 3.6 Based on Hamilton scale score analyzed in PG

#### 3.6.1 Abundance difference analysis

According to the above principle of abundance analysis, we found that after the alpha statistical analysis on the PG, each index of PG 2 was larger than PG 1, but there was no statistical significant difference (*p* > .05).

#### 3.6.2 Based on metastas analysis

The metastas method was used to analyze the two groups at the Class, Family, Genus, and Order levels. We concluded that *Betaproteobateria* (2.4805% vs. 5.0373%, *p* = .007) had significant differences between the two groups in Class.

In Family, Eubacteriaceae (<0.1% vs. <0.1%, *p* = .002), Alcaligenaceae (2.462% vs. 5.016%, *p* = .013), Peptostreptococcaceae (<0.1% vs. 0.188%, *p* = .015), Comamonadaceae (<0.1% vs. <0.1%, *p* = .015), Thermoanaerobacteraceae (<0.1% vs. <0.1%, *p* = .03) had significant differences between the two groups.

In Genus, *Catenibacterium* (<0.1% vs. 0.119%, *p* = .001), *Slackia* (<0.1% vs. <0.1%, *p* = .001), *Cloacibacillus* (<0.1% vs. <0.1%, *p* = .001), *Eubacterium* (<0.1% vs. <0.1%, *p* = .002), *Comamonas* (<0.1% vs. <0.1%, *p* = .007), *Ezakiella* (<0.1% vs. <0.1%, *p* = .009), *Senegalimassilia* (<0.1% vs. <0.1%, *p* = .009), *Gordonibacter* (<0.1% vs. <0.1%, *p* = .01), *Romboutsia* (<0.1% vs. 0.1768%, *p* = .01), *Gelria* (<0.1% vs. <0.1%, *p* = .02), *Sutterella* (1.003% vs. 2.5546%, *p* = .04), *Faecalibacterium* (6.1537% vs. 3.7208%, *p* = .047), *Anaerostipes* (0.2178% vs. <0.1%, *p* = .048) had significant differences between the two groups.

In Order, Burkholderiales (2.4805% vs. 5.0373%, *p* = .02) and Thermoanaerobacterales (<0.1% vs. <0.1%, *p* = .03) had significant difference between two groups.

**FIGURE 4:**
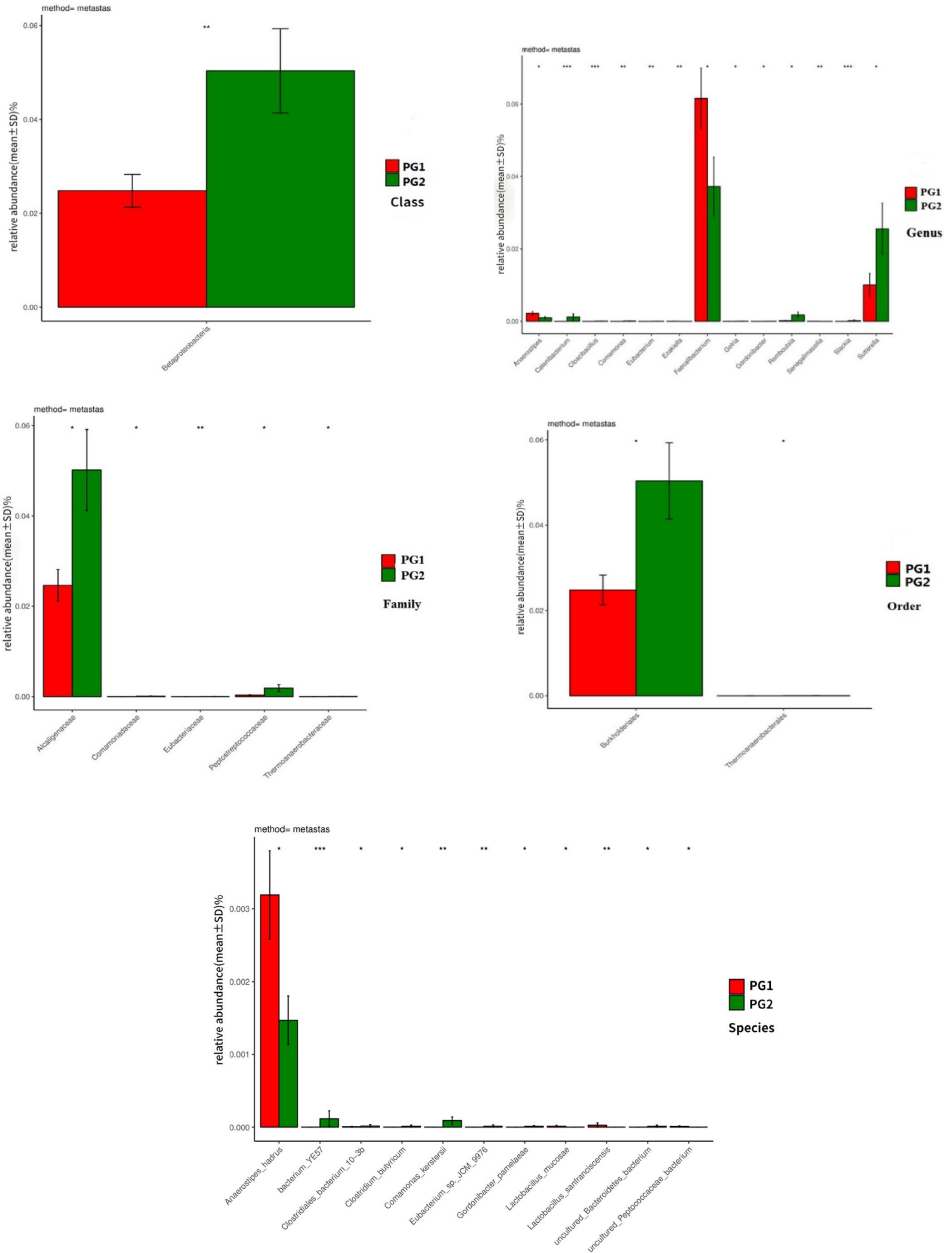
The variability of the two groups at different levels; the abscissa is the Species of bacteria, and the ordinate is mean ± *SD*

The Species results are not listed here; the detailed results are shown in Figure 4. As above mentioned, because the accuracy of the data is extremely low for bacterial groups with the relative abundance of less than 0.1% in MiSeq analysis, we summarized the flora with abundance greater than 0.1%, as shown in Table 6.

**TABLE 6:** Detailed results of differential analysis between PG1 and PG2

	OUT	Mean (PG2)%	Mean (PG1)%	*p*‐Value	*p*.signif
Class	Betaproteobacteria	0.050373	0.024805	.006993	**
Family	Alcaligenaceae	0.050162	0.024624	.012987	*
	Peptostreptococcaceae	0.00188	<0.001	.014985	*
Genus	*Catenibacterium*	0.001192	<0.001	.000999	***
	*Sutterella*	0.025546	0.01003	.041958	*
	*Faecalibacterium*	0.037208	0.061537	.047952	*
	*Anaerostipes*	<0.001	0.002178	.048951	*
Order	Burkholderiales	0.050373	0.024805	.018981	*
Species	*Anaerostipes_hadrus*	0.001471	0.003191	.021978	*

Notes.

The relative abundance of 0.1% or more in at least one group,* indicates that it is relevant Multiple * indicates that it is more relevant.

#### 3.6.3 Based on LEfSe analysis

**FIGURE 5:**
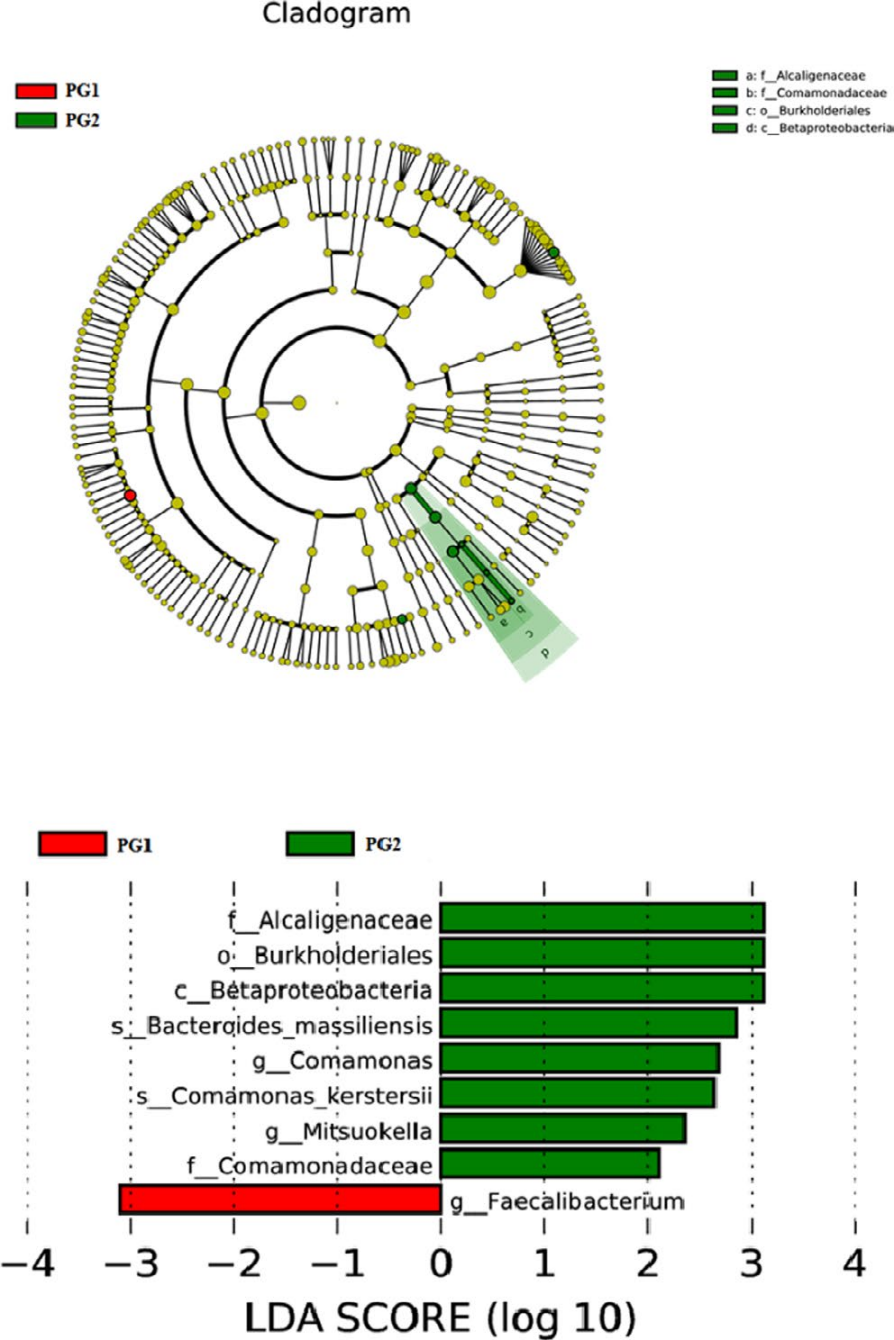
Distribution of dominant microflora in LDA SCORE SCORE (see Figure 3 for annotations)

As can be seen in Figure 5, based on the LDA SCORE, we found that the dominant bacterial communities were obtained between the two groups according to the HAMA anxiety SCORE: Alcaligenaceae, Burkholderiales, Betaproteobacteria, and *Facecalibacterium* in the two groups.

## 4 DISCUSSION

### 4.1 Differences between PG and HG

Through sequencing data and an alpha diversity analysis, we found that there was no statistical difference in the abundance of each group. The results were the same as those observed by Naseribafrouei et al. (2014), but different from those of Jiang et al. (2015). Now most scholars believe that the higher the diversity of intestinal flora, the better the health (Matsuoka & Kanai, 2015). For example, in intestinal inflammatory disease, the diversity of intestinal flora decreases significantly, and diversity increases with the improvement of the disease (De Angelis et al., 2013). However, other studies have pointed out that there is no significant difference in the diversity of intestinal flora between autistic patients and healthy people. Therefore, the specific mechanism of microbial diversity in depressed patients still needs to be further explored. In Jiang's study (Jiang et al., 2015), all the participants recruited were <40 years old and had no history of hypertension. In this study, we limited the age to 65 years old but did not count evaluate hypertension history. In addition, there were also large differences in regional dietary habits, which may also be the reason for difference in the results.

In this study, the imbalance of intestinal flora is mainly reflected by changes in five levels: Class, Family, Genus, Order, and Species. Compared with healthy individuals, the differences are mainly reflected in Rikenellaceae, *Alistipes* (*Alistipes* belongs to the Rikenellaceae Family) in PG, there was a significant difference between the PG and the HG in the bacterial group with a relative abundance of 0.1% or more at least one group (Table 5), the abundance of which were increased significantly. Alistipes is an indole‐positive bacterium, which can break down tryptophan to produce indole, and affects human tryptophan metabolism. Since tryptophan is a precursor of 5‐HT, the rise of Alistipes may affect the intestine tryptophan metabolism, which affects brain neurotransmitter signaling (Song et al., 2006). On the other hand, some studies indicate that the abundance of Alistipes is detected in patients with chronic fatigue syndrome, and chronic fatigue syndrome is often accompanied by the development of anxiety and depression. Therefore, the effect of *Alistipes* on 5‐HT metabolism in patients with depression is worth studying.

The abundance of Lachnospiraceae, Succinivibrionaceae, *Alloprevotella*, *Anaerostipes*, *Aeromonadales*, and *Succinivibrio*, which belong to the thick‐walled bacteria with a relative abundance of 0.1% or more at least one group, was significantly reduced (Table 5). Prior studies (Wong et al., 2016) have pointed out that cysteine‐1 inhibitors may have antidepressant effects. Studies have found that the changes of Lachnospiracea abundance are consistent with changes of the microbiome in cysteine‐1‐deficient mice. In addition, some studies (Duncan et al., 2007) suggest that Lachnospiraceae is involved in the metabolism of short‐chain fatty acids (SCFAs). SCFAs are an important source of intestinal epithelial cell energy, which can also affect the permeability of intestinal epithelial cells and various biochemical reactions (Vince et al., 1990; Wong et al., 2006). The abundance of Lachnospiraceae may affect intestinal permeability, which may induce neurotoxic substances, neurotransmitters, etc., and affect the brain's function through the vagus nerve more easily. In addition, SCFAs can also speed up the secretion of 5‐HT and accelerate gastrointestinal motility. Therefore, we suspect that the decrease of Lachnospiraceae abundance may also be related to gastric motility disorders in patients with depression.

By LEfSe software analysis, we found that *Barneslella* (inflammatory) was the dominant flora in PG, which is different from the results of Jiang's study (Jiang's results were Porphyromonadaceae, *Alistipes* (inflammation)), but it may be related to inflammatory bacteria. In HG, Lachnospiraceae and *Alloprevotella* are the dominant flora. The abundance of Lachnospiraceae in depression patients is reduced. We also demonstrated the importance of Lachnospiraceae in the human body.

### 4.2 Difference of bacteria group in PG by HAMA classification

Because depression was accompanied by anxiety, we classified the depression group into anxiety group and nonanxiety group according to the HAMA score and as the PG1 and PG2. According to alpha diversity analysis, there was no significant difference in bacterial diversity and abundance between PG1 and PG2. However, the metastas analysis revealed that the proportion of Betaproteobateria (Proteobacteria), Alcaligenaceae (Inflammatory), Peptostreptococcaceae, *Catenibacterium*, *Sutterella*, Burkholderiales in PG2 (which was significantly higher than PG1), and *Anaerostipes*, *Anaerostipes‐hadrus* (inflammatory), and *Faecalibacterium* was significantly lower than in PG1 (Table 6). We found that the levels of inflammatory bacteria with anxiety symptoms in depression were significantly lower than those without anxiety. In addition, we also found that *Faecalibacterium* was higher in patients with anxiety. Existing research shows that *Faecalibacterium* has a strong anti‐inflammatory effect and the lack of *Faecalibacterium* can lead to the onset of Crohn's disease (Barrett et al., 2012; Sokol et al., 2008). When this bacterium was transplanted in animals, it was found to be able to fight against colitis, and if combined with human immune cells, it could produce anti‐inflammatory effects (Barrett et al., 2012; Sokol et al., 2008).

In addition, by LEfSe analysis, the dominant groups of PG2 were Alcaligenaceae, Burkholderiales, Betaproteobacteria, and the PG1 was *Facecalibacterium*. Therefore, by means of grouping, it was found that the level of inflammation in patients without anxiety symptoms was higher than in patients with anxiety symptoms; the microflora varied between the two groups.

In summary, although we found a large number of changes in bacterial abundance in the study, a large amount of literature confirmed that not only did inflammatory factors increase, but also inflammatory responses increased in patients with depression. However, we found that there was neither an increase nor decrease in the anti‐inflammatory flora in our study. It is speculated that age may not be strictly controlled and segmented, and cardiovascular disease may not be strictly controlled in participants. In addition, the samples might not be strictly aseptically handled when collected, and there may be a risk of sample contamination.

### 4.3 Limitations

This study is a cross‐sectional study, which only evaluates the difference between the two groups, and there are constraints, such as the culturability of the bacteria, which have not further verified the importance of flora we selected. More experimental data are still needed. The score scale also has a certain degree of concealment during the assessment process. In addition, we also found that the influencing factors were relatively high using PCA measurement (PC1 37.67%, PC2 20.18%). Therefore, it is necessary to further improve the clinical observation indicators and related influential factors in subsequent studies to improve the accuracy of the experiment.

## 5 CONCLUSION

In this study, a comprehensive analysis of the intestinal micro‐ecology of patients with depression was performed by high‐throughput sequencing of 16sRNA. It was found that there was a significant difference in intestinal micro‐ecology between patients and healthy volunteers, which was manifested in the proportion of pathogenic bacteria and *Alistipes,* which increased, Lachnospiraceae and other beneficial bacteria were significantly reduced. The analysis revealed that *Barnesiella* was the dominant group in PG, and Lachnospiraceae and *Alloprevotella* were the dominant group in HG. This study uses clinical samples of first‐episode depression, and its findings provide theoretical and practical basis for the prevention and treatment of depression, and the regulation of bacterial flora (such as Alistipes and Lachnospiraceae found in the article) to cure or prevent depression in the future, which has a strong practical significance for the occurrence and development of clinical treatment of depression. In addition, we found that the patients with depression with anxiety symptoms and those without anxiety symptoms also have large differences in flora, which will be more useful for our clinical judgment of accompanying symptoms and it will be helpful for accompanying symptoms treatment. The experimental results have enriched the correlation between depression and intestinal flora.

## CONFLICTS OF INTERESTS

All authors report no conflicts of interest.

## AUTHOR CONTRIBUTION

SJZ, YQW, and ZRW conceived the original idea for this study. The study design was planned by SJZ, ZRW, and FDY and with support from WDW and QZ. SJZ and WDW prepared the manuscript with repeated revisions commented on and amended by ZRW and YBZ. All authors were involved in the interpretation of the results. We would like to thank Dr. ZR Wang and FD Yang for his guidance in this paper. We would also like to thank Editage (www.editage.cn) for English language editing.

### Peer Review

The peer review history for this article is available at https://publons.com/publon/10.1002/brb3.2036.

## Data Availability

Data were made available to all interested researchers upon request.
